# Hypergravity load-induced hyperglycemia occurs due to hypothermia and increased plasma corticosterone level in mice

**DOI:** 10.1186/s12576-022-00844-2

**Published:** 2022-08-01

**Authors:** Chikara Abe, Chikako Katayama, Kazuhiro Horii, Bakushi Ogawa, Kento Ohbayashi, Yusaku Iwasaki, Fumiaki Nin, Hironobu Morita

**Affiliations:** 1 Department of Physiology, Gifu University Graduate School of Medicine, 1-1 Yanagido, Gifu, 501-1194 Japan; 2 Laboratory of Animal Science, Graduate School of Life and Environmental Sciences, Kyoto Prefectural University, 1-5 Hangi-cho, Shimogamo, Sakyo-ku, Kyoto, 606-8522 Japan

**Keywords:** Gravity, Vestibular system, Adrenaline, Noradrenaline

## Abstract

Hypothermia has been observed during hypergravity load in mice and rats. This response is beneficial for maintaining blood glucose level, although food intake decreases. However, saving glucose is not enough to maintain blood glucose level during hypergravity load. In this study, we examined the contribution of humoral factors related to glycolysis in maintaining blood glucose level in a 2 G environment. Increased plasma corticosterone levels were observed in mice with intact peripheral vestibular organs, but not in mice with vestibular lesions. Plasma glucagon levels did not change, and decrease in plasma adrenaline levels was observed in mice with intact peripheral vestibular organs. Accordingly, it is possible that increase in plasma corticosterone level and hypothermia contribute to prevent hypoglycemia in a 2 G environment.

## Introduction

Gravity is a stressor that affects physiological homeostasis. All living organisms have evolved in a 1 G environment on Earth. In the future, we plan to go to space, including Moon and Mars, which have a gravitational environment different from that of Earth. To survive in other gravitational environments, an understanding of the mechanisms of gravity-dependent physiological adaptation is required. Some experiments in this field have been conducted using a hypergravity environment, because a sustained reduced gravity smaller than 1 G is difficult to create on Earth. Adaptation of physiological responses, including the cardiovascular system [1–3], autonomic nervous system [1, 3], and muscle–skeletal system [4, 5] have been examined using a hypergravity environment.

Metabolic adaptation is also important for survival in a hypergravity environment. Energy expenditure is increased, because loading size increases in the hypergravity environment; thus, food intake needs to be increased to maintain blood glucose. However, a decrease in body weight accompanied by hypophagia is observed in the first few days of hypergravity loading in rats and mice [6, 7]. Subsequently, an increase in both food intake and body weight is observed after adaptation to the hypergravity environment; no animal has died during hypergravity load. In this physiological process, it is unclear how blood glucose level is maintained in the first few days during hypergravity load, although food intake becomes almost zero, and energy demand increases. Exposure to a hypergravity environment also induces a decrease in body temperature (BT) and activity [6, 8–10]. This hypothermic response might contribute to the maintenance of blood glucose level by reducing glucose usage. We previously showed that gravitational change induces an increase in sympathetic nerve activity via the peripheral vestibular organs [3, 11], suggesting that the catecholaminergic system contributes to the maintenance of blood glucose level. However, the response is transient, and gravitational change-induced sympathoexcitation is not sustained during a 2 G load. Accordingly, other factors, especially humoral factors related to glycolysis, may be involved in maintaining blood glucose in a hypergravity environment. To test this hypothesis, we examined changes in humoral factors, including plasma corticosterone, glucagon, insulin, and catecholamine, induced by a 2 G load for 2 h, using intact and lesioned vestibular mice.

## Materials and methods

The animals used in this study were maintained in accordance with the Guiding Principles for Care and Use of Animals in the Field of Physiological Science, as set by the Physiological Society of Japan. The experiments were approved by the Animal Research Committee of Gifu University. Male (*n* = 24) and female (*n* = 20) C57BL/6 J mice weighing 20–26 g were purchased from Japan SLC.

**Fig. 1:**
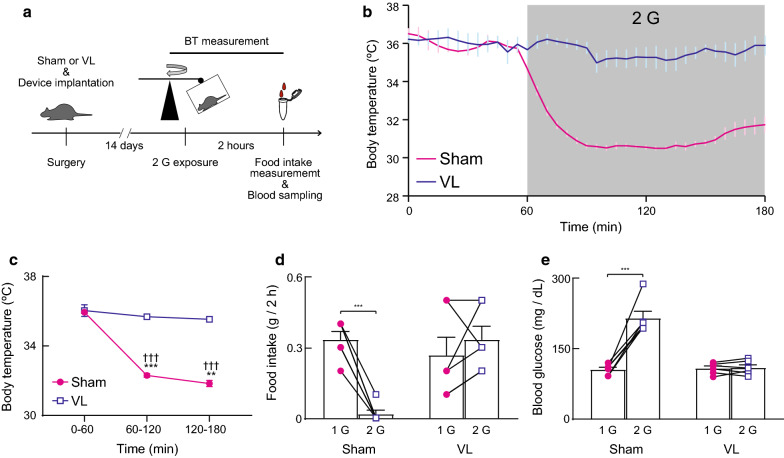
**A** Timeline of the experiment. **B** Changes in body temperature (BT) during 2 G exposure in vestibular lesion (VL, *n* = 4) or sham-operated (Sham, *n* = 4) mice. **C** Summarized data of changes in BT during 2 G exposure in sham (*n* = 4) and VL (*n* = 4) mice. The symbol “*” indicates comparisons *vs*. the 0–60 min timepoint; “†” indicates comparisons *vs*. VL. Double or triple significant symbols indicate *P* < 0.01 or *P* < 0.001, respectively. **D**–**E** Summarized data of changes in food intake (**D**) and blood glucose (**E**) 2 h after an exposure to the 2 G environment in sham (*n* = 6) and VL (*n* = 6) mice. Triple significant symbol indicates *P* < 0.001

We first examined whether hypergravity load-induced hypothermia was reproducible, as in a previous study [6]. Mice (*n* = 8) were intraperitoneally anesthetized with a mixture of ketamine (120 mg/kg) and xylazine (12 mg/kg) to induce vestibular lesions (VL). This study also included sham-operated mice (Sham). After careful removal of the tympanic membrane, malleus, incus, and stapes, labyrinthine fluid was aspirated. An agent for vestibular lesions (Periodon, Neo Dental International) was placed through the oval window. Conversely, while the tympanic membrane was removed during sham VL surgery, the auditory ossicles remained intact. The implantable programmable device for the measurement of BT and activity was used in our experiment (nano tag^®^, Kissei Comtec Co., Ltd). The device was implanted into the abdominal cavity of the mice. After surgery, the mice received postoperative boluses of atipamezole (an α2-adrenergic antagonist, 2 mg/kg, s.c.), penicillin G potassium (3000 U/kg, s.c.), and ketoprofen (4 mg/kg, s.c.). The mice were housed in groups of four per cage under a 12:12 h light:dark cycle. The temperature was maintained at 24 ± 1 °C. 2 weeks after the surgery, the measurement time and sampling rate (5 min intervals) were programmed using a non-contact IC card. To expose the mice to a 2 G environment, centrifugation using a gondola-type rotating box with a 40-cm-long arm was employed (Takei Scientific Instruments Co., Ltd). All mice were provided food (CE-2, CLEA Japan) and water ad libitum. All the data saved in the device before and during the 2 G load were obtained using the same non-contact IC card (Fig. 1A).

We next examined changes in food intake and blood glucose under 2 G load for 2 h in sham and VL mice (Fig. 1A). The experiment was conducted 2 weeks after the sham and VL operations. In the 2 G group, food intake and blood sampling were conducted immediately after the cessation of loading (11:00 am). There was a 48-h interval in the measurement of food intake and blood glucose in the 1 G and 2 G environment. Blood was collected from a small tail cut under isoflurane inhalation to measure glucose levels (Glucose PILOT, Technicon). In the other mice, blood (100 μL) was collected from the ophthalmic artery using a glass tube (Fisherbrand Microhematocrit Capillary Tubes, Fisher Scientific) under isoflurane inhalation to measure humoral factors in the plasma. The blood was centrifuged (4000 rpm for 10 min) to obtain the plasma, which was measured for glucagon (YK091, Yanaihara), corticosterone (EC3001-1, ASSAYPRO), and insulin (AKRIN-011S, Fujifilm) using commercial ELISA kits. Since the measurement of plasma catecholamine requires a larger volume of blood than that of humoral factors, additional mice were used. To measure plasma catecholamine levels, plasma was added to 10% sodium disulfide (final concentration, 2%) and 3,4-dihydroxybenzylamine (5 pmol; as an internal standard). Plasma catecholamines were purified using activated alumina and measured using high-performance liquid chromatography with electrochemical detection (HPLC–ECD).

All data sets were checked for normality using either the D’Agostino–Pearson omnibus normality or Kolmogorov–Smirnov test. Equal variances were successively examined using the Brown–Forsythe test. If the criteria of normality and equal variance were satisfied, statistical significance was evaluated using the *t* test or two-way ANOVA. As necessary, Tukey’s or Bonferroni’s multiple comparison tests were applied. All values are expressed as the mean ± the standard error of the mean, and statistical significance was set at *P* < 0.05.

## Results

A previous study from our laboratory demonstrated that a decrease in BT of mice was observed during exposure to a 2 G environment, and this response was absent in mice with VL [6]. The response was reproduced in this study (Fig. 1B, C). The BT decreased from 36 ± 0.1 ℃ to 31.6 ± 0.1 ℃ 60 min after initiation of the 2 G load in sham mice. On the other hand, the BT did not change in VL mice (36.1 ± 0.3 ℃ to 35.5 ± 0.5 °C).

Food intake was significantly decreased for 2 h in sham mice at a 2 G load (Fig. 1D), whereas euphagia was observed in the VL mice. Although hypophagia was observed in sham mice, blood glucose levels were significantly increased (Fig. 1E). This response was not observed in VL mice.

**Fig. 2:**
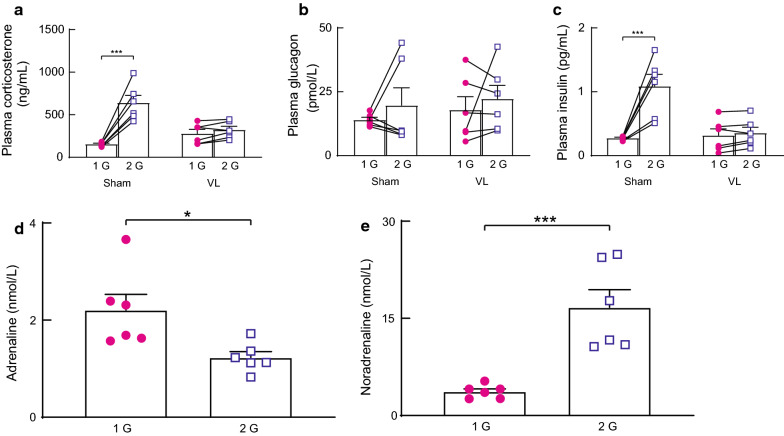
**A**–**C** Summarized data of changes in plasma corticosterone **A**, glucagon **B**, and insulin **C** levels during 2 G load for 2 h, in the vestibular lesion (VL, *n* = 6) and its corresponding sham-operated (Sham, *n* = 6) mouse. Statistics: two-way ANOVA with Tukey’s multiple comparisons test. Triple significant symbol indicates *P* < 0.001. **D**–**E** Summarized data of changes in plasma adrenaline **D** and noradrenaline **E** levels, 2 h after an exposure to a 2 G environment in 1 G (*n* = 6) and 2 G (*n* = 6) mice. Single or triple significance symbols indicate *P* < 0.05 or *P* < 0.001, respectively

Plasma corticosterone and insulin levels were significantly increased in sham mice under 2 G load, while plasma glucagon levels did not change (Fig. 2A–C). In VL mice, no humoral factors, including corticosterone, glucagon, and insulin, were altered. In addition, plasma adrenaline level was significantly decreased, but plasma noradrenaline level was significantly increased under 2 G load (Fig. 2D, E).

## Discussion

The major findings of this study are as follows: 1) blood glucose level was increased under 2 G load, although food intake was decreased; 2) plasma corticosterone and insulin levels were increased under 2 G load, but plasma glucagon level was unchanged; and 3) plasma adrenaline level was decreased, but plasma noradrenaline level was increased under 2 G load. 

Plasma corticosterone and noradrenaline might have contributed to the increased blood glucose level under the 2 G environment in this study. The response of the plasma corticosterone might be specific to the 2 G load-induced activation of the peripheral vestibular organs, because the response was not observed in VL mice. Furthermore, a previous study from our laboratory demonstrated that 2 G load-induced activation of corticotropin releasing hormone (CRH) in the paraventricular hypothalamic nucleus (PVN) was suppressed in VL rats [12]. Accordingly, it is possible that the suppressed glucocorticoid response in VL mice might be due to a decreased response of CRH neurons in the PVN. Plasma corticosterone is known to be involved in glycolysis, which increases blood glucose level. Glucocorticoids promote gluconeogenesis in the liver, whereas in the skeletal muscle and white adipose tissue, they decrease glucose uptake and utilization by antagonizing the insulin response [13]. However, the effect of noradrenaline on glycolysis remains unclear. β2 adrenergic receptors in the liver and muscle participate in glycolysis [14], suggesting that plasma noradrenaline contributes to increase in blood glucose level. However, the affinity of noradrenaline for β2 adrenergic receptors is weaker than that of adrenaline; thus, the effect of increasing blood glucose level might be small. It has been reported that noradrenaline has no effect on the increase in blood glucose level, whereas adrenaline does [15]. Taken together, the increase in plasma corticosterone level and hypothermia may be indispensable for the prevention of hypoglycemia in a 2 G environment.

In the present study, plasma insulin was increased by the 2 G load in sham mice but not VL mice. It is possible that the increased plasma insulin is due to increased blood glucose by 2 G load. Furthermore, as previously reported [15], the increase in plasma noradrenaline level during 2 G load might contribute to an increase in the plasma insulin level. Increases in blood glucose and plasma insulin seem to be effective for metabolism in the peripheral organs. However, previous studies reported decreases in both body mass and activity in 2 G load [6, 7], suggesting the lack of the system for glucose usage in the cells. Furthermore, in the present study, 2 G load induced hypothermia. Given the gradual decreases in body mass, activity, and body temperature during long-term hypergravity load [6, 7], the responses of blood glucose and plasma insulin might be one of the processes involved in adaptation to the new gravitational environment via the vestibular system.

Hypergravity is a gravitational stress, and the autonomic nervous system is recruited to maintain physiological homeostasis. C1 neurons in the medulla oblongata are known to be one of the centers regulating the autonomic nervous system, which respond to stressors. Previous studies from our laboratory demonstrated that stimulation of peripheral vestibular organs using a hypergravity load induced c-Fos expression in C1 neurons [6, 11]. C1 neurons are involved in metabolic responses, and selective activation of C1 neurons using chemogenetic tools can increase food intake and blood glucose and plasma corticosterone levels in rats and mice [16, 17]. Accordingly, it is possible that C1 neurons participated in the response observed in the present study. However, several studies have indicated that adrenal sympathetic nerves that activate adrenergic chromaffin cells are derived from C1 neurons [18, 19]. Furthermore, the adrenal glands are indispensable for the increase in blood glucose level caused by the selective activation of C1 neurons; this could be due to the fact that the descending projection from the C1 neurons to the adrenal gland is necessary for glycogenolysis [17]. Since the present study showed a decrease in plasma adrenaline level, some populations of C1 neurons on the adrenergic pathway might not be recruited under a 2 G load.

In the present study, 2 G load-induced hypothermia was suppressed by VL, suggesting that hypothermia is induced through the peripheral vestibular organ. An intracerebral injection of adrenaline induces hypothermia in mice [20]. This finding suggests that activation of C1 neurons, one of the adrenergic neurons in the medulla oblongata, might induce hypothermia in 2 G environment. Furthermore, the selective activation of C1 neurons using the optogenetics tool decreased the sympathetic nerve activity to the brown adipose tissue through the α2 adrenergic receptors in the rostral raphe pallidus area [21]. Since 2 G-induced c-fos expression in C1 neurons can be significantly suppressed by VL [6, 11], the hypothermia response might be due to activation of the pathway between peripheral vestibular organs and C1 neurons.

In the present study, decreased food intake was observed in addition to 2 G load-induced hypothermia. Both hypophagia and hypothermia are observed in motion sickness mediated by the vestibular system [22]. It is difficult to determine whether mice experience motion sickness, because they show no emetic responses. However, allotriophagy, an index of motion sickness, has been observed in rats under hypergravity conditions [23, 24]. Response to changes in BT are also related to the peripheral vestibular organs. Shaking induced by hypothermia was suppressed in mice lacking the α9 acetylcholine receptor subunit, which is predominantly expressed in vestibular hair cells [25]. Accordingly, it is suggested that the response to both hypothermia and hypophagia is due to motion sickness via the peripheral vestibular organs.

The 2 G load-induced hypothermia observed in this study might be attributed to a decrease in plasma adrenaline level. Symptoms of motion sickness, including cold sweating and pallor, are known to be induced by sympathoexcitation [26], which seems to contradict the data presented here. This might be attributed to the pattern of vestibular inputs, that is, phasic or tonic inputs, since there are some reports that electrical stimulation of the vestibular nerve elicits a sympathetic nervous response, including excitation, inhibition, or a combination of both [27]. On the other hand, tonic vestibular inputs, such as hypergravity (3 G), increased renal sympathetic nerve activity in rats at the onset of loading; however, the value returned to the baseline level 3 min later [1]. Since short-term (4.5 s) microgravity exposure also induces sympathoexcitation [28], it is thought that sympathoexcitation might occur through the peripheral vestibular organ in response to changes in vestibular inputs (phasic changes in gravity). In other words, sympathoinhibition and lack of sympathoexcitation might be seen in the case of long-term tonic vestibular inputs, which is supported by previous studies [29, 30]. On the other hand, the increase in plasma noradrenaline level might be due to activation of noradrenergic chromaffin cells of the adrenal medulla if the sympathoexcitation was not sustained during the 2 G load. These possibilities, including changes in plasma adrenaline and noradrenaline level induced by a 2 G load, should be examined in future studies.

## Conclusions

Blood glucose levels should be maintained in a hypergravity environment, because the energy demand is increased. The hypothermic response and increase in plasma corticosterone level might contribute to prevent hypoglycemia in a 2 G environment. On the other hand, changes in plasma catecholamine including adrenaline and noradrenaline might not contribute to maintain blood glucose in a 2 G environment.

## Data Availability

The data underlying this article will be shared on reasonable request to the corresponding author.
